# The Impact of Substance Use Disorder and Drug Transfer into Breast Milk: Implications for Maternal and Infant Health

**DOI:** 10.3390/pharmaceutics17060719

**Published:** 2025-05-29

**Authors:** Yongzong Yang, Bofang Yi, Tao Zhang

**Affiliations:** 1 Meinig School of Biomedical Engineering, Cornell University, 121A Weill Hall, Ithaca, NY 14853, USA; yy2353@cornell.edu; 2 School of Pharmacy and Pharmaceutical Sciences, SUNY-Binghamton University, 96 Corliss Ave, Johnson City, NY 13790, USA; byi@binghamton.edu

**Keywords:** breast milk, substance use disorder, lactation, drug transfer, pharmacokinetics, relative infant dose, milk-to-plasma ratio, infant health, breastfeeding safety

## Abstract

Breast milk provides significant health benefits to both infants and mothers, offering protection against infections and enhancing cognitive development. This paper examines the complex effects of substance use disorder (SUD) during pregnancy and lactation, focusing on the pharmacokinetics of drug transfer into breast milk. It highlights the mechanisms by which drugs enter milk, emphasizing the roles of passive diffusion and active transport, particularly through breast cancer resistance protein (BCRP). The study explores the impact of various substances on fetal and infant health, with a focus on the relative infant dose (RID) and milk-to-plasma (MP) ratio as key metrics for assessing drug safety in breastfeeding. The findings underscore the need for careful evaluation of maternal drug use during lactation to balance the benefits of breastfeeding with potential risks.

## 1. Why Is Milk Important?

In evaluating the safety of drug use during lactation, it is crucial to highlight the numerous benefits that breast milk offers to both mother and infant [1,2,3,4]. The advantages of breastfeeding have been primarily derived from observational cohort studies and epidemiological research, which provide substantial evidence of a causal relationship between breastfeeding and health benefits, as demonstrated by their consistent and observable associations [5]. For instance, infants who are breastfed have a lower susceptibility to infections, a benefit attributed to the presence of anti-infective agents such as secretory IgA, lactoferrin, lysozymes, and defensins in breast milk [6,7]. This protective effect is particularly significant in preterm infants, where the incidence of necrotizing enterocolitis is notably decreased if they are breastfed [8,9]. Additionally, breastfed infants tend to exhibit superior cognitive development compared to those who are formula-fed. This is supported by findings from various cohort studies and the PROBIT trial, which indicated that breastfeeding correlates with an increase in IQ by approximately 8 points at ages 6–7, reflecting a meaningful impact on cognitive outcomes [10,11].

Moreover, experimental studies in mice suggest that breast milk may play a role in the infant’s immunological development. Maternal cytokines and dietary allergens in milk contribute to the infant’s immune system development by fostering tolerance to external antigens, effectively preparing the infant for post-weaning environmental exposures [12,13]. This highlights the mammary gland’s potential function as an information-processing organ that equips the infant for future challenges. There are likely other critical biological functions of breast milk yet to be uncovered [2,14]. Considering these extensive benefits, pediatric guidelines strongly advocate for exclusive breastfeeding during the first six months and continued breastfeeding thereafter. Therefore, when assessing the risks of maternal drug use during breastfeeding, it is essential to carefully weigh these health benefits against any potential need to discontinue breastfeeding [4].

We conducted a narrative literature review using the PubMed, Scopus, and Google Scholar databases, focusing on studies published in English between 2000 and 2024. We included peer-reviewed clinical studies, case reports, systematic reviews, and pharmacokinetic data relevant to drug transfer into human breast milk; studies examining the impact of maternal substance use (both prescribed and illicit) during pregnancy and lactation; and data on relative infant dose (RID), milk-to-plasma (M/P) ratio, and infant safety outcomes.

We excluded studies on substances no longer in clinical use, animal-only studies without human correlation, and articles lacking quantitative or safety-relevant data.

## 2. Mechanism of Drug Secretion into Breast Milk

Drug transfer into breast milk involves complex interactions between the tissue structures of the mammary gland and various transport mechanisms. The mammary gland consists of epithelial cells forming ducts and alveoli, essential for milk production and secretion [15]. These alveoli are surrounded by myoepithelial cells, which contract in response to the hormone oxytocin, facilitating milk ejection [16]. During pregnancy, the mammary gland undergoes significant development [15]. Progesterone stimulates the proliferation of ductal structures, while prolactin promotes the growth of lobuloalveolar structures necessary for lactation [17]. This development creates an intricate network where drugs can interact with the mammary epithelial cells.

Drugs can enter breast milk through several mechanisms, including passive diffusion, facilitated diffusion, and active transport [18]. Passive diffusion depends on the concentration gradient between maternal plasma and milk, allowing lipophilic and non-ionized drugs to move freely. Facilitated diffusion involves carrier proteins that aid in the movement of specific molecules across cell membranes [19].

**Figure 1:**
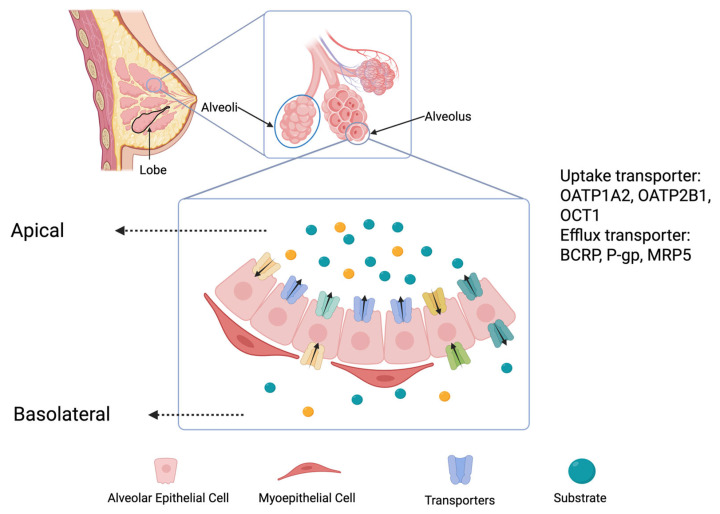
Layered anatomical and molecular structure of the lactating mammary gland and key transporters involved in drug transfer. The mammary gland exhibits a hierarchical organization from lobes to alveoli to individual alveolar epithelial cells. Several membrane transporters regulate the movement of substances between maternal plasma and milk. Uptake transporters—including organic anion transporting polypeptide 1A2 (OATP1A2), organic anion transporting polypeptide 2B1 (OATP2B1), and organic cation transporter 1 (OCT1)—facilitate substrate entry into mammary epithelial cells. Efflux transporters—breast cancer resistance protein (BCRP), P-glycoprotein (P-gp), and multidrug resistance-associated protein 5 (MRP5)—mediate active transport of compounds into milk. These transporter systems collectively influence drug disposition in breast milk and potential infant exposure. (Created in BioRender. Yi, B. (2025) https://BioRender.com/o2moqhl).

Several transporters play a crucial role in drug transfer into milk (Figure 1). These include the efflux transporter breast cancer resistance protein (BCRP) and P-glycoprotein (P-gp) [20,21]. BCRP is a key efflux transporter highly expressed in tissues with barrier functions, such as the blood–brain barrier, placenta, and intestines [21,22]. In the mammary gland, BCRP expression is regulated by the prolactin–JAK2/STAT5 pathway [23]. This transporter actively pumps various substances, including drugs and toxins, into milk [24]. BCRP has a broad substrate range, including phytochemicals, food toxins, and medications like cimetidine, acyclovir, and methotrexate [25,26]. It also plays a vital role in transporting essential nutrients, such as vitamin B2 (riboflavin), into milk. This function is critical for ensuring that nursing infants receive the necessary vitamins, but it also means that drugs can exploit BCRP to enter milk. Factors influencing drug transfer into milk include the drug’s ionization state, plasma protein binding, and lipophilicity. The acidic environment of milk compared to that of plasma causes basic drugs to ionize, trapping them in milk. Drugs with low plasma protein binding and high lipophilicity are more likely to concentrate in milk. These characteristics, along with BCRP substrate specificity, determine the milk-to-plasma drug concentration ratio (MP ratio), affecting how substances are transferred to the infant through breastfeeding. A quantitative parameter to estimate the drug amount that enters the infant is the relative infant dose (RID) [27].

## 3. Substance Use Disorder (SUD) in Pregnancy

Substance use disorder (SUD) is a complex condition characterized by an uncontrolled use of hazardous substances, despite harmful consequences [28,29]. Individuals with SUD experience an intense tendency to use certain substances, such as alcohol, tobacco, or drugs, which leads to significant impairment or distress [30,31,32]. This disorder affects the brain’s reward, motivation, and memory systems, making it challenging for individuals to control their impulses and cravings [33,34]. Over time, SUD can lead to a range of physical and mental health issues, impacting personal relationships, employment, and overall quality of life [28]. The severity of SUD can vary, ranging from mild to severe, and this disorder often requires comprehensive treatment strategies, including behavioral therapies and medication-assisted treatment, to support recovery and prevent relapse [35]. In this paper, alcohol, stimulants, cannabis, sedatives, and tobacco will be discussed.

Although the primary focus of this paper is breastfeeding, it is necessary to address substance use disorder (SUD) during pregnancy, as prenatal exposure to harmful substances significantly impacts neonatal outcomes and maternal behavior postpartum. Moreover, continued substance use after delivery increases the risk of drug transfer into breast milk, warranting a comprehensive overview of this process that begins with prenatal substance exposure.

It is important to distinguish between medically supervised prescription drug use and substance use disorder (SUD). SUD, as defined by the DSM-5, is based on behavioral patterns, including loss of control over substance use, continued substance use despite harm, and cravings. The therapeutic use of prescribed medications, even during pregnancy, does not constitute SUD in the absence of such features [29]. Epidemiological surveys estimate that 10–15% of pregnant women report alcohol consumption, 5–8% report cannabis use, and 0.5–1% report the use of illicit drugs during pregnancy [28,36]. While exact quantities are difficult to determine due to variability in self-reporting, patterns range from occasional use to daily or binge consumption.

When SUD occurs during pregnancy, it presents unique challenges and risks for both the mother and the developing fetus [36,37]. Substance use can adversely affect fetal development, leading to complications such as low birth weight, preterm birth, and developmental disorders [38,39]. Different substances pose varying risks; for example, alcohol use can result in fetal alcohol spectrum disorders [40], while opioid use can lead to neonatal abstinence syndrome [41], where the newborn experiences withdrawal symptoms after birth. Additionally, pregnant individuals with SUD may face barriers to accessing prenatal care, stigmatization, and a lack of social support, all of which can exacerbate their health outcomes [42]. Hence, it is important to explore SUD-related-drugs’ impact on maternal milk and infants.

In this review, we summarize several major classes of substances that may contribute to SUD, including sedatives, stimulants, alcohol, cannabis, and tobacco. We focus on the presence of these substances and their metabolites in breast milk, examining their potential effects on infants and the implications for maternal and infant health. Given the extensive discussion on opioids in the existing literature, this class is not included in our review.

## 4. Sedatives in Pregnancy and Breastfeeding

For managing acute anxiety or insomnia, the use of sedatives by breastfeeding mothers should ideally be limited to short durations, typically less than 2–3 days, particularly in unsupervised outpatient settings. This is crucial, unless close monitoring of the infant’s condition is feasible, due to the potential for adverse effects such as sedation and respiratory issues [43]. The RID for most sedatives is generally low, usually not more than 3%, except for phenobarbital, for which it can be higher [44]. Avoiding their prolonged use is important to prevent drug accumulation in the infant, especially given the variability in individual drug clearance, which is often unpredictable. Limiting sedative use to a few days is prudent for managing acute symptoms. This cautious approach is necessary due to the limited safety data on long-term sedative use during breastfeeding.

The following sections will describe some of the commonly used sedatives and their implications during breastfeeding. Key findings from representative studies are summarized in a table for quick reference, highlighting differences in clinical contexts between short-term anxiety management and other conditions.

### 4.1. Diazepam

Diazepam is a benzodiazepine, acts as an anxiolytic, muscle relaxant, and anticonvulsant, and is widely used in various clinical settings [45]. Diazepam is extensively metabolized in the liver by CYP2C19 and CYP3A4 into active metabolites such as nordiazepam, temazepam, and oxazepam [46,47]. These metabolites contribute to its prolonged pharmacological effects, with a long half-life leading to their potential accumulation. Diazepam is excreted into breast milk, and both diazepam and its active metabolite nordiazepam can accumulate in the serum of breastfed infants with repeated dosing. Due to their long half-lives, timing breastfeeding around dosing does not significantly reduce exposure. A safety scoring system suggests a cautious use during breastfeeding [27]. Studies indicate that high doses, such as those during surgical procedures, may increase the risks of infant weight loss and hyperbilirubinemia [27]. Other agents are preferred, especially for newborns or preterm infants. After a single dose, breastfeeding can typically resume without delay, but a cautious approach would suggest waiting 6 to 8 h for newborns or preterm infants. During long-term use, infants should be monitored for sedation, poor feeding, and weight gain issues.

The maternal levels of diazepam and its metabolites were monitored in several studies. The average RID of diazepam is near 3%, which can be considered as a safe value. Three patients given 10 mg of diazepam orally three times daily for 6 days postpartum had average milk levels of diazepam plus nordiazepam of 79 mcg/L after 4 days and 130 mcg/L after 6 days [48]. In a separate case, a 1-day postpartum woman had a colostrum diazepam level of 100 mcg/L 25 h after the last of three intravenous 5 mg doses [49]. Four women given 10 mg of diazepam at bedtime for 6 days, starting 3 days postpartum, had milk levels ranging from 17 to 39 mcg/L for diazepam and from 19 to 52 mcg/L for nordiazepam [50]. One woman on an irregular regimen of 6 to 10 mg daily of oral diazepam had breastmilk levels of 27 to 164 mcg/L of diazepam plus nordiazepam between 9 days and 3.5 months postpartum [51]. A benzodiazepine-abusing woman on 80 mg of diazepam and 30 mg of oxazepam daily had milk levels of diazepam and nordiazepam averaging 185 and 307 mcg/L and 124 and 141 mcg/L, respectively, on days 14 and 15 and 200 and 158 mcg/L and 140 and 85 mcg/L, respectively, on days 23 and 25 during maternal intake of 30 mg of diazepam daily. Diazepam and nordiazepam levels in milk dropped to 67 mcg/L and 42 mcg/L, respectively, on day 30, during the intake of 10 mg of diazepam daily, and were for both drugs 6 mcg/L nine days after discontinuing diazepam [52]. Eight women receiving intravenous diazepam (2.5 to 10 mg) during surgical sterilization had no detectable diazepam or nordiazepam in their breastmilk (<150 mcg/L), with an estimated maximum systemic exposure of 3% for their infants [53].

Three infants breastfed from birth while their mothers took 10 mg of diazepam 3 times daily showed no lethargy or hypoventilation during a 6-day observation period, although there was concern that nordiazepam might compete with bilirubin for hepatic glucuronide conjugation in neonates [48]. Another infant, whose mother took 10 mg of diazepam orally 3 times daily beginning on day 5 postpartum, experienced weight loss, lethargy, and an EEG consistent with sedative effect when 8 days old, likely due to diazepam or its metabolites in breastmilk. In a study of eight infants breastfed from birth during maternal diazepam therapy (dosages unspecified), three had mild jaundice in the first few days postpartum, though this was not considered unusual [54]. Sedation was reported in a breastfed infant when the mother on 6 to 10 mg of diazepam daily nursed within a few hours of taking a dose, but not if nursing occurred 8 or more hours after a dose [53]. In a telephone follow-up study of 124 mothers who took benzodiazepines while nursing, about 10% used diazepam, and none reported sedation in their infants [55]. Some mothers in a longitudinal study who took diazepam discontinued breastfeeding due to infant drowsiness [56]. A retrospective chart review of 298 mothers and infants born at 37 weeks or beyond indicated that infants whose mothers received 0.1 mg/kg or greater of diazepam during tubal ligation had a 9-fold increased risk of significant weight loss and a 3-fold increased risk of hyperbilirubinemia requiring phototherapy, compared to those whose mothers received lower doses [57].

Diazepam can be used during breastfeeding with caution, balancing the need for maternal treatment against potential infant exposure. Monitoring and considering alternative medications may be appropriate, especially for vulnerable infants. Professionals should guide mothers on safe practices and closely observe infants for any signs of adverse effects.

### 4.2. Phenobarbital

Phenobarbital is metabolized primarily in the liver through various pathways. The primary enzyme involved in its metabolism is CYP2C9, although other enzymes such as CYP2C19 and CYP2E1 can also play roles [58,59]. Phenobarbital is converted into several metabolites, but the major metabolic pathway involves the hydroxylation of the phenobarbital molecule, resulting in the formation of p-hydroxyphenobarbital. This metabolite is then conjugated with glucuronic acid to form a glucuronide conjugate, which is excreted in the urine [58].

In published reports of anticonvulsant use during breastfeeding, the maternal dosage of phenobarbital and its concentration in breast milk can be highly variable due to the influence of other co-administered anticonvulsants. Some, like phenytoin and carbamazepine, stimulate the metabolism of phenobarbital, while others, like valproic acid, inhibit it [60]. This variability complicates the calculation of the weight-adjusted percentage of maternal dosage. For instance, in some reports, the RID value ranged from 39% to 135%, and this huge variation makes the RID not ideal to analyze phenobarbital [44].

In women taking phenobarbital for 3 days, its average milk levels at 23 h post-dose were as follows: 90 mg daily (0.85 mg/L; range, 0.8 to 1 mg/L), 150 mg daily (1.25 mg/L; range, 1 to 1.5 mg/L), and 225 mg daily (5.2 mg/L; range, 2.7 to 5 mg/L) [61]. The same research reported that phenobarbital milk levels in two women taking 125 mg three times daily along with phenytoin ranged from 5.6 to 6 mg/L at various times of the day postpartum. Another report showed a milk phenobarbital level of 2.7 mg/L in a patient taking 30 mg four times daily for 3.5 days [49]. Several studies have documented the effects of phenobarbital in breastfed infants. An infant whose mother was taking a combination of phenobarbital, primidone, phenytoin, and sulthiame had serum phenobarbital levels of 2 mg/L at 17 days and 12.7 mg/L at 1 month of age [62]. Another study found that serum phenobarbital concentrations in infants increased by about 2 to 5 mg/L for each mg/kg of maternal dose, with higher increases in the first 5 days postpartum, likely due to transplacental passage [63].

Case reports have highlighted adverse effects, including two 1-week-old infants who exhibited deep slumber and difficulty awakening potentially due to phenobarbital in breast milk [64]. Another infant exhibited extreme sedation and methemoglobinemia likely caused by a combination of phenobarbital and phenytoin [65]. An infant death from overlying and suffocation was possibly contributed to by sedation from phenobarbital, primidone, and phenytoin in breast milk [66]. Additionally, a breastfed infant experienced probable drug withdrawal symptoms, manifested as spontaneous tremors, after the abrupt discontinuation of breastfeeding by a mother taking phenobarbital [67]. Another case involved an infant with drug-induced drowsiness and poor weight gain, which led to the discontinuation of breastfeeding [68]. Conversely, a breastfed infant whose mother was on a combination of phenobarbital, primidone, and carbamazepine did well initially but developed seizures after abrupt weaning, which were controlled with continued phenobarbital administration [69].

While most studies on phenobarbital during breastfeeding focus on short-term or perioperative exposure, clinical scenarios often involve chronic administration, particularly for seizure disorders. The chronic maternal use of phenobarbital has been associated with sustained and sometimes elevated drug levels in breast milk, leading to measurable serum concentrations in infants. Several case reports document adverse outcomes including persistent drowsiness, poor feeding, and delayed weight gain in breastfed infants [63]. In some cases, abrupt weaning led to withdrawal symptoms such as tremors or seizures, indicating physiological dependence. Additionally, when phenobarbital is used alongside other antiepileptic drugs, their interactions may enhance drug accumulation or toxicity in the infant. These findings highlight the importance of individualized risk assessment, regular infant monitoring (e.g., serum levels, weight gain, neurobehavioral development), and consideration of alternative therapies or feeding strategies during long-term phenobarbital treatment.

These findings underscore the importance of closely monitoring infants breastfed by mothers taking phenobarbital, given the potential for significant drug exposure and adverse effects. Further research is needed to better understand the safety profile of phenobarbital during breastfeeding.

### 4.3. Haloperidol

Haloperidol is an antipsychotic medication used to treat a variety of psychiatric conditions. It is metabolized in the liver primarily by the enzyme CYP3A4, with contributions from CYP2D6 and, to a lesser extent, CYP1A2 [70]. The major metabolic pathways include reduction to its major metabolite, reduced haloperidol, oxidation to form various metabolites, and conjugation (e.g., glucuronidation), with excretion occurring primarily in the urine and, to some extent, in the feces [71]. When considering haloperidol use during breastfeeding, it is important to note that the drug is excreted into breast milk in small amounts. While the concentration in breast milk is generally lower than the maternal plasma concentration, the exact amount can vary. The small amounts of haloperidol that pass into breast milk can potentially cause side effects in the nursing infant, such as sedation, developmental delays, or extrapyramidal symptoms (movement disorders). However, the risk of significant adverse effects in infants is considered low, especially if the mother is on a low to moderate dose of haloperidol. Based on available clinical studies and case reports, this typically refers to a daily oral dose of 2–10 mg, which has been associated with low levels of drug transfer into breast milk and minimal adverse effects in breastfed infants. Doses above this range, particularly ≥20 mg/day, have shown increased variability in milk concentrations and may carry a higher risk of infant sedation or extrapyramidal symptoms.

The maternal levels of haloperidol in breast milk vary widely based on dosage and adherence. Milk levels have been reported from undetectable to as high as 988 mcg/L, with variability often attributed to assay methods and undetected metabolites [72]. For example, a woman taking an average dose of 29.2 mg daily had a milk level of 5 mcg/L, while another on 12 mg had had a milk level of 2 mcg/L [73]. Random levels during a 5 mg twice-daily regimen ranged from 18 to 23.5 mcg/L, later reducing to 4 mcg/L [74]. In contrast, nonadherence led to lower levels. Infant exposure is generally low, with urine levels around 1.5 mcg/L observed in some cases [75].

Infant outcomes during maternal haloperidol therapy are generally positive, with normal growth and developmental milestones reported in most cases. For instance, one infant breastfed for six weeks, whose mother was on a dose of 5 mg twice daily, showed normal development at 6 and 12 months [74]. However, caution is advised, as a small study noted developmental declines in some infants exposed to combination therapies [75]. Excessive sedation and poor feeding were reported in an infant when haloperidol was combined with other medications, highlighting the need for careful monitoring and dosage management [75].

Haloperidol use during breastfeeding should be approached with caution. While many infants exhibit normal development, the potential for adverse effects, particularly when haloperidol is combined with other drugs, necessitates careful monitoring.

### 4.4. Dichloralphenazone

Dichloralphenazone is a sedative and hypnotic medication often used in combination with other drugs to treat tension and migraine headaches. It is metabolized in the liver through reduction and oxidation pathways. Dichloralphenazone is broken down into chloral hydrate and, subsequently, trichloroethanol and trichloroacetic acid, which are then excreted in the urine [76]. When considering its use during breastfeeding, dichloralphenazone can pass into breast milk in small amounts. Potential effects on the nursing infant include sedation and respiratory depression. Although the risk is low, careful monitoring of the infant and consultation with a professional are recommended if the mother requires this medication.

Dichloralphenazone, a combination of chloral hydrate and antipyrine, shows variable milk levels [77]. In a study, a woman taking 1.3 g daily (equivalent to about 830 mg of chloral hydrate) had trichloroethanol milk levels between 1.3 and 3.2 mg/L [78]. Antipyrine, given separately to lactating women, reached peak milk levels of 10 to 30 mg/L approximately 10 min post-ingestion, with a milk half-life averaging 11.6 h. The reported milk concentrations of lorazepam range from approximately 1.6 to 35 mcg/L, depending on maternal dosage and timing postpartum. The relative infant dose (RID) of lorazepam has been estimated at approximately 8.5% of the maternal weight-adjusted dose, which is within the generally accepted safety threshold of <10% [79]. Despite the moderate RID, no significant sedation has been reported in most case series and observational studies of breastfed infants, though rare reports of transient drowsiness exist—particularly when lorazepam is combined with other CNS depressants.

In a documented case, minimal morning sedation was observed in a 5-month-old infant whose mother consumed 1.3 g of dichloralphenazone nightly, alongside chlorpromazine. Despite this, the infant’s development was normal at 3 months. Trichloroethanol was detectable in the infant’s plasma 21 h after maternal ingestion, indicating some level of exposure through breastfeeding [78].

Dichloralphenazone use during breastfeeding should be approached with caution due to potential infant sedation. Due to its short half-life and metabolism via glucuronidation (resulting in inactive metabolites), lorazepam is often preferred over diazepam for use in lactating women. Although the developmental effects seem minimal, monitoring for sedation and other side effects is advisable.

### 4.5. Fospropofol

Fospropofol is a prodrug of propofol used as a sedative–hypnotic for the induction and maintenance of general anesthesia or sedation. It is metabolized in the liver by alkaline phosphatase enzymes to release propofol, which then undergoes further metabolism primarily through glucuronidation and hydroxylation by the CYP2B6 and CYP2C9 enzymes [80]. The metabolites are excreted in the urine. During breastfeeding, fospropofol’s metabolite, propofol, can pass into breast milk in small amounts. The potential effects on the nursing infant include sedation and respiratory depression, though the risk is minimal. Breastfeeding mothers should be observed, monitoring these effects in their infants and consulting professionals for advice.

Fospropofol is rapidly metabolized to propofol, which has been studied in breastfeeding contexts. Analysis of the maternal levels showed that propofol is present in colostrum after anesthesia, with peak levels varying based on dosage and infusion duration. For instance, a woman receiving 0.42 mg/kg/minute had her colostrum levels drop from 1.53 mg/L to 0.12 mg/L over 8 h. Another woman receiving a continuous infusion reached a peak of 4.91 mg/L [81]. Single doses prior to cesarean sections resulted in average levels of 0.17 mg/L at 4 h and 0.14 mg/L at 8 h post-dose. Despite these levels, it is estimated that infants receive about 0.2% of the maternal dose, which is unlikely to affect healthy, term infants [82]. For continuous infusions, concentrations drop substantially within 8–12 h post-infusion. The estimated infant exposure is minimal, usually less than 0.2% of the maternal weight-adjusted dose.

In studies, breastfeeding infants showed no sedation when their mothers received propofol as part of anesthesia. However, the milk output post-surgery was reduced, possibly due to stress and fluid restriction [83]. Some cases reported milk discoloration, likely due to propofol or other medications. There were no significant differences in the immediate breastfeeding rates between different anesthesia methods, though the long-term breastfeeding rates were lower with general anesthesia. Overall, while propofol presence in breast milk is minimal, monitoring infant response and milk production is advised.

Fospropofol, through its metabolite propofol, appears safe for short-term use during breastfeeding, with minimal transfer to milk. However, careful monitoring of infants is recommended to ensure safety, especially considering potential impacts on milk production and any unusual milk discoloration.

### 4.6. Alprazolam

Alprazolam is a benzodiazepine used primarily to treat anxiety and panic disorders. It is metabolized in the liver by CYP3A4 into active metabolites such as alpha-hydroxyalprazolam and inactive metabolites [84]. These metabolites are excreted in the urine. Alprazolam can pass into breast milk in small amounts. Potential effects on a nursing infant include sedation, feeding difficulties, and respiratory issues. While the risk is generally low, it is essential to monitor the infant for adverse effects and consider alternative treatments if necessary.

Alprazolam is present in breast milk at low concentrations. In a study [85] with eight lactating women who received a single 0.5 mg dose of alprazolam, the milk levels peaked at an average of 3.7 mcg/L around 1.1 h post-dose. The half-life in milk was approximately 14.5 h, with no metabolites found. An exclusively breastfed infant would receive about 0.5 to 5 mcg/kg daily, roughly 3% of the maternal dose. Another study reported milk levels of 5.42 mcg/L at 1 month postpartum with a 1 mg daily dosage. Higher doses, such as 2.4 mg daily, resulted in levels up to 24.5 mcg/L [86]. These data suggest that infant exposure is minimal, yet monitoring is advised.

Infant reactions to alprazolam in breast milk can include irritability, especially following maternal discontinuation, indicating potential withdrawal symptoms [87]. In studies, some infants exposed to alprazolam showed drowsiness, though these reactions generally did not require medical intervention [88]. Alprazolam may increase serum prolactin, potentially affecting lactation. Cases of galactorrhea and amenorrhea have been reported with high doses, but these resolved after discontinuation [89]. While the prolactin rise may not impact established lactation, cautious use and monitoring are recommended to ensure infant safety and maternal health.

Alprazolam can be used during breastfeeding at low doses, but with careful monitoring of infant sedation or withdrawal symptoms. Consultation is essential to balance maternal treatment needs and infant safety.

### 4.7. Propofol

Propofol is a widely used intravenous anesthetic agent for the induction and maintenance of general anesthesia and sedation. It is metabolized in the liver primarily through glucuronidation and hydroxylation by the CYP2B6 and CYP2C9 enzymes [80]. The metabolites are excreted in the urine. Propofol passes into breast milk in small amounts. The primary concern for nursing infants is sedation and respiratory depression, but the risk is considered low. Breastfeeding mothers using propofol should be monitored, and professional should be consulted to ensure the safety of the infant.

Propofol was studied in breastfeeding women undergoing anesthesia. In women receiving 0.42 mg/kg/minute, the colostrum levels peaked at 1.53 mg/L and decreased to 0.12 mg/L over 8 h [81]. Another case reported 4.91 mg/L at 30 min with a lower infusion rate of 0.18 mg/kg/minute for 6 h. After a single 2.5 mg/kg bolus, the colostrum levels averaged 0.17 mg/L at 4 h and 0.14 mg/L at 8 h. Continuous infusion after a bolus showed varied levels: 0.33 mg/L at 4 h and 0.036 mg/L at 6 h [82]. For postpartum women receiving a single dose, it was shown that their infants would receive about 0.0052 mg/kg, representing 0.2% of the maternal dosage [83]. Propofol was quantified at 24 mcg/L in one case of milk discoloration. Two mothers had levels of 0.13 mg/L and 2.78 mg/L at 90 min, dropping to 0.84 mg/L by 300 min after dosing [90].

No sedation was observed in infants breastfed 1.5 to 5 h post-extubation after maternal propofol use [90]. The milk output was reduced post-surgery, likely due to surgical stress and fluid restrictions [83]. Studies indicate that general anesthesia with propofol may delay lactation initiation [91]. Milk discoloration occurred in several cases, potentially linked to propofol or its metabolites [92,93,94]. Propofol anesthesia did not significantly affect the short-term breastfeeding rates, but the long-term rates were lower than with spinal anesthesia.

Propofol appears in breast milk in low concentrations and is present for a limited time post-administration. Its impact on breastfed infants is minimal, with no sedation noted in short-term observations. While propofol may temporarily affect milk output and delay lactation initiation, it is generally considered safe for breastfeeding mothers.

### 4.8. Carisoprodol

Carisoprodol is a muscle relaxant used to relieve the pain and discomfort associated with acute musculoskeletal conditions. It is metabolized in the liver primarily by CYP2C19 into its active metabolite, meprobamate, which is also a sedative and an anxiolytic [95]. Both carisoprodol and meprobamate are excreted in the urine. Carisoprodol can pass into breast milk and may cause sedation, hypotonia, and feeding difficulties in nursing infants. The risk is moderate, and alternative treatments should be considered. If carisoprodol is necessary, close monitoring of the infant is essential, and consultation with a professional is recommended.

Carisoprodol is primarily metabolized to meprobamate, which contributes to its sedative effects. With a half-life of 4 h for carisoprodol and 10 h for meprobamate, a breastfeeding mother taking 700 mg three times daily showed trough breastmilk levels averaging 0.9 mg/L for carisoprodol and 11.6 mg/L for meprobamate. The minimum estimated infant dose was 1.9 mg/kg daily, or 4.1% of the maternal dosage. Extrapolating to average doses, the infant might receive 3 mg/kg daily or 6% of the maternal dose [96]. In another case, carisoprodol and meprobamate were detectable in breastmilk for two days post-hospitalization, and only meprobamate on the third day [97]. A separate study showed breastmilk concentrations of 1.4 mg/L for carisoprodol and 10.9 mg/L for meprobamate 2 h after dosing, decreasing to 0.8 mg/L and 17.1 mg/L, respectively, 3.5 h post-dose. The maximum infant intake was estimated at 2.7 mg/kg daily, or 6.9% of the maternal dosage [98].

An exclusively breastfed infant had unmeasurable serum concentrations of carisoprodol (<2 mg/L) and meprobamate (<4 mg/L) postpartum, suggesting low exposure or potential metabolic adaptation from in utero exposure [96]. One mother taking carisoprodol with propoxyphene and acetaminophen noticed no adverse reactions in her partially breastfed infant, who developed normally by 6 months. However, slight sedation was observed in a newborn during the first month when the mother took 700 mg of carisoprodol four times daily while exclusively nursing [98].

Carisoprodol and its metabolite meprobamate appear in breast milk at levels that could potentially sedate an infant, although significant adverse effects are uncommon. Monitoring is advised, especially for signs of sedation in exclusively breastfed infants. The decision to use carisoprodol while breastfeeding should weigh the benefits to the mother against potential risks to the infant, with alternative treatments considered when appropriate.

### 4.9. Lorazepam

Lorazepam is a benzodiazepine used to treat anxiety disorders, insomnia, and seizures. It is metabolized in the liver primarily through glucuronidation to form an inactive glucuronide conjugate, which is excreted in the urine [99]. Lorazepam passes into breast milk in small amounts and may cause sedation, feeding difficulties, and respiratory issues in nursing infants. The risk is relatively low, but careful monitoring of the infant is necessary. Breastfeeding mothers should consult with professionals to weigh the benefits and risks and consider alternative treatments if appropriate.

Lorazepam levels in breast milk were studied in various contexts. Four women taking 3.5 mg orally before cesarean sections had colostrum levels averaging 8.5 mcg/L at 4 h post-dose [100]. Another woman on 2.5 mg twice daily had milk levels of 12 mcg/L (free) and 35 mcg/L (conjugated) on day 5 postpartum. The total drug level suggests that an exclusively breastfed infant would receive about 7 mcg/kg daily, or 8.5% of the maternal dosage [101]. Another case showed that a mother taking 2.5 mg 1–3 times daily and lormetazepam had milk levels of 123 mcg/L on day 5, decreasing to 55 mcg/L by day 7 [102]. Three women taking 0.5 mg daily had peak milk levels ranging from 1.64 to 1.98 mcg/L, with trough levels often below 0.5 mcg/L [103].

Infants exposed to lorazepam through breastfeeding generally showed no signs of sedation. A newborn whose mother took 2.5 mg twice daily for 5 days post-delivery showed no sedation [101]. In a study of 124 mothers taking benzodiazepines, including lorazepam, none reported infant sedation [55]. However, sedation was noted in one infant exposed to a combination of risperidone and lorazepam, which resolved after discontinuing lorazepam [104].

Lorazepam is present in breast milk at varying levels depending on dosage and frequency. While generally considered safe for breastfeeding mothers, with minimal adverse effects on infants, monitoring for sedation is advised, especially in cases of polypharmacy. The decision to use lorazepam should involve weighing maternal benefits against potential infant risks, with alternatives considered if necessary.

## 5. Tobacco in Pregnancy and Breastfeeding

Tobacco use during breastfeeding is concerning due to nicotine and other harmful chemicals transferring into breast milk [105]. These substances can lead to an increased risk of respiratory problems, developmental delays, and altered sleep patterns in infants [106]. While the relative infant dose (RID) of nicotine is relatively low, the cumulative effects of tobacco exposure can be significant. It is strongly recommended to avoid smoking and seek cessation support. If cessation is not possible, strategies such as smoking after breastfeeding and avoiding exposure to secondhand smoke can help mitigate the risks. Prioritizing a smoke-free environment is crucial for the infant’s health.

### Nicotine

Nicotine is a potent alkaloid found primarily in tobacco plants and is well known for its stimulating effects on the central nervous system [107]. Upon consumption, nicotine is rapidly absorbed into the bloodstream and metabolized primarily in the liver by cytochrome P450 enzymes, resulting in various metabolites, including cotinine, which has a longer half-life than nicotine itself [108]. This metabolic process is significant when considering the implications for breastfeeding mothers who smoke or use nicotine replacement therapies, such as transdermal patches. Studies have shown that nicotine and cotinine can transfer into breast milk, which potentially exposes breastfeeding infants to these substances [106]. The levels of nicotine in breast milk can vary based on the maternal smoking habits and the usage of nicotine patches, with concentrations being notably higher during active smoking. This transfer of nicotine may pose risks to infants, including potential impacts on development, behavior, and health outcomes, such as an increased risk of sudden infant death syndrome (SIDS). Therefore, understanding the metabolism of nicotine and its implications for lactation is crucial for guiding breastfeeding mothers in making informed decisions regarding nicotine use.

In a study involving fifteen lactating women who smoked an average of 17 cigarettes daily (ranging from 14 to 20), researchers investigated the nicotine levels in breast milk during smoking and following smoking cessation while using nicotine transdermal patches. The patches were administered in decreasing doses of 21 mg, 14 mg, and 7 mg daily. One participant who smoked six cigarettes daily began on a 14 mg patch. Milk samples were collected before and after nursing, approximately 2 to 3 weeks apart, for the analysis of nicotine and its metabolite, cotinine. The findings indicated that while smoking, the average nicotine concentration in breast milk was around 200 mcg/L. During the use of the 21 mg patch, milk nicotine concentration was approximately 175 mcg/L, which was statistically similar to the levels observed during smoking. Conversely, lower patch doses resulted in reduced nicotine concentrations, with the 14 mg and 7 mg patches yielding about 140 mcg/L and 70 mcg/L, respectively. The corresponding cotinine levels also decreased with lower doses. The calculated daily nicotine equivalent dosages for infants were as follows: 25.2 mcg/kg with smoking, 23 mcg/kg with the 21 mg patch, 15.8 mcg/kg with the 14 mg patch, and 7.5 mcg/kg with the 7 mg patch. On average, infants ingested approximately 1.9% of the maternal weight-adjusted nicotine dosage and about 7.8% when cotinine was included in the calculations.

Maternal smoking has been identified as a significant risk factor for sudden infant death syndrome (SIDS), with nicotine believed to play a critical role by diminishing the dopamine levels in the carotid bodies, thereby impairing an infant’s ability to respond to hypoxic conditions. Additionally, nicotine present in breast milk may adversely affect male infants by reducing heart rate variability. In a separate study of nine breastfed infants, whose mothers were using a 21 mg nicotine patch for smoking cessation, the average plasma cotinine concentration in the infants was found to be 22 mcg/L (ranging from 19 to 25 mcg/L), which constituted about 13.4% of the maternal cotinine levels. Furthermore, maternal nicotine use could impact lactation, as cigarette smoking is known to reduce milk yield, potentially due to nicotine’s effect on lowering the serum prolactin levels. In a group of 15 nursing mothers using nicotine patches, it was observed that their average milk production was 17% lower than the average literature values, although the study did not make direct comparisons between smokers and nonsmokers. Notably, the infant milk intake while using the nicotine patch was comparable to that during active smoking.

The data suggest that while nicotine replacement therapy, such as transdermal patches, may lower the nicotine levels in breast milk compared to smoking, breastfeeding mothers who use these patches still transfer nicotine and its metabolite, cotinine, to their infants. Given the potential risks associated with nicotine exposure in infants—including SIDS and developmental concerns—caution is advised. Mothers should consult professionals to weigh the benefits and risks of nicotine use during breastfeeding, as the safety of infant exposure to nicotine, even at reduced levels, remains a critical concern.

## 6. Stimulants in Pregnancy and Breastfeeding

Stimulants are a diverse class of substances that elevate central nervous system (CNS) activity, resulting in increased alertness and energy, mood elevation, and sometimes euphoria. This group includes amphetamines, cocaine, nicotine (tobacco), alcohol, and cannabis (marijuana)—each with unique pharmacodynamic profiles but overlapping neurochemical mechanisms, primarily involving dopamine, norepinephrine, and serotonin pathways. Amphetamines and cocaine are prototypical CNS stimulants that directly enhance the synaptic concentrations of catecholamines, leading to pronounced psychostimulant effects and high abuse potential. Nicotine, the active component of tobacco, similarly stimulates nicotinic acetylcholine receptors and indirectly promotes dopaminergic transmission, reinforcing its addictive properties and stimulating cardiovascular and CNS activity. Notably, both alcohol and cannabis demonstrate biphasic dose–response characteristics. At lower doses, these substances can act as stimulants, producing effects such as reduced social inhibition, mild euphoria, or heightened sensory perception. However, at moderate to high doses, they primarily exert depressant effects—slowing neural activity, impairing coordination, and inducing sedation or lethargy. This duality is particularly relevant when evaluating their impact on maternal behavior and infant exposure during breastfeeding, as the clinical presentation of substance use can vary significantly with dose and individual metabolism. Including these substances under the stimulant umbrella reflects their initial CNS activation effects and aligns with classifications used in many clinical and pharmacological frameworks, while still acknowledging their complex, dose-dependent actions.

### 6.1. Sulpiride

Sulpiride is a psychotherapeutic agent and galactagogue that is not FDA-approved in the United States but is used in other countries for increasing milk production. While it has shown effectiveness in increasing milk volume in mothers with documented low milk production [109], its benefit appears limited to cases with complete absence of initial milk production [110]. Sulpiride should not replace proper evaluation and counseling on breastfeeding techniques and modifiable factors affecting milk production. It is excreted into breast milk in significant amounts, and the dosage that infants receive exceeds the commonly accepted limit of 10% of the maternal weight-adjusted dosage [111]. Despite the high milk levels, two studies reported no adverse effects on breastfed infants during its short-term use [112,113]. However, the postpartum depression risk and sulpiride’s potential to cause depression make it unsuitable for mothers with a history of major depression or those requiring prolonged treatment. The side effects in nursing mothers include tiredness, headaches, and leg edema.

Sulpiride undergoes minimal hepatic metabolism, and its clearance is primarily renal, with a significant portion excreted unchanged in the urine. Unlike many other psychotropic drugs, sulpiride is not extensively metabolized by the cytochrome P450 enzyme system. However, some hydroxylation occurs, catalyzed by CYP3A4, and the drug undergoes conjugation reactions including glucuronidation. This unique metabolic profile contributes to its relatively low plasma protein binding and predictable pharmacokinetics. In one study, sulpiride was undetectable in milk after maternal doses of up to 200 mg daily, but the detection limit was not provided. In 20 women taking 50 mg twice daily, the milk concentrations ranged from 260 to 1970 mcg/L, translating to an average infant dosage of 8.7% (range 2–18%) of the maternal weight-adjusted dose [114]. Single-dose studies showed that the milk concentrations peaked at 10 to 293 mcg/L depending on the dose, with calculated infant doses of 1.2 to 46.3 mcg/day [111]. One postpartum woman treated with 100 mg daily had milk concentrations of 445.8 and 192.2 mcg/L at 7.5 and 11.5 h post-dose, respectively [115].

A study of 14 women treated with sulpiride at a dose of 50 mg three times daily for four weeks reported no side effects in their breastfed infants. The mothers in this study experienced increased serum prolactin levels, which facilitated milk production. However, the study did not evaluate the long-term infant health outcomes, leaving gaps in understanding the broader implications of sulpiride use during lactation [113]. In another study involving 24 nursing mothers who received the same sulpiride dosage for two weeks, no adverse effects were observed in the infants. This study further supports the safety of short-term sulpiride use as a galactagogue, but like the prior study, it lacks data on prolonged use and infant serum drug levels [112].

In a placebo-controlled trial of 28 women within four months postpartum, sulpiride was administered at 50 mg three times daily for four weeks. The participants in the treatment group showed significantly higher infant weight gain compared to those in the placebo group by the end of the study (1081 vs. 795 g). The serum prolactin levels rose in the sulpiride group, while the placebo-treated participants experienced a slight decrease. However, the study noted no correlation between increased prolactin levels and milk production, raising questions about the mechanisms behind the observed weight gain. Importantly, most infants in both groups received supplemental feedings, which made it unclear whether the weight gain in the sulpiride group was due to increased milk production or to other factors [113].

A 90-day study of 66 primiparous mothers compared sulpiride (100 mg three times daily for four days, then 50 mg three times daily) to a placebo. Women receiving sulpiride maintained elevated serum prolactin levels throughout the study, while the placebo-treated mothers experienced a natural decline. Infant weight gain differences were observed only up to day 15, with no significant differences thereafter. This study suffered from a high dropout rate of 38%, which limits the robustness of its conclusions [116]. Another randomized, double-blind trial examined 60 women 25 to 40 days postpartum. The participants received l-sulpiride, d-sulpiride, d,l-sulpiride, or a placebo for 15 days. Milk production increased in all drug groups, with women who initially produced no milk being able to breastfeed exclusively after 10 to 15 days of sulpiride therapy. However, milk production declined after drug discontinuation, and the study lacks data on lactation education or nursing frequency, leaving key factors unexamined [117].

Sulpiride increases the serum prolactin levels, promoting galactorrhea at higher rates compared to other psychotropic drugs [117,118,119,120,121]. Several studies have demonstrated its efficacy in enhancing milk production, but many have significant design flaws, including lack of randomization and inadequate blinding [112,113,114,116,117,122,123]. Sulpiride’s effects on the prolactin levels were evident in some studies, with treated mothers maintaining elevated baseline levels compared to those receiving a placebo. Despite these findings, the correlation between prolactin levels and milk yield remains inconsistent, and long-term safety data for infants are lacking.

Sulpiride has shown promise as a galactagogue in mothers with low milk production, particularly in cases of complete absence of milk at baseline. While its short-term use appears safe for infants, significant gaps remain regarding its long-term effects and the mechanisms behind its efficacy. Its ability to increase the serum prolactin levels highlights its potential utility, but inconsistent correlations with milk yield and the risk of maternal side effects, including depression, limit its broader application. Further randomized, blinded studies with robust designs are needed to establish its safety and effectiveness in diverse lactating populations [112,113,114,116,117].

### 6.2. Castor Oil

Castor oil, derived from the seeds of the castor plant (Ricinus communis), contains triglycerides primarily composed of ricinoleic acid esters. While castor oil is a well-known stimulant laxative, traditional uses in some cultures claim it as a galactagogue to promote lactation [124,125]. For example, in parts of India, castor oil is applied to the breasts, or castor leaves are used as poultices to stimulate milk production [126]. However, no scientifically valid clinical trials support the use of castor oil or its preparations as galactagogues. Furthermore, castor beans and some derivatives can be toxic, containing ricin and ricine, which are removed during oil purification [127]. Due to a lack of evidence on their safety and efficacy, galactagogues such as castor oil should never replace evaluation and counseling on modifiable factors affecting milk production [109,110].

No data exist regarding the excretion of castor oil components into breast milk or their direct effects on nursing infants. However, ricinoleic acid, the active component, is thought to have limited intestinal absorption, which may reduce its systemic effects. Despite this, other cathartics with established safety profiles are recommended for nursing mothers.

Castor oil is hydrolyzed in the small intestine by pancreatic lipase, releasing ricinoleic acid, the compound responsible for its laxative effect. Ricinoleic acid acts locally in the intestine to stimulate peristalsis by interacting with EP3 prostanoid receptors, which modulate smooth muscle contractions. Because the systemic absorption of ricinoleic acid is minimal, its presence in maternal serum and breast milk is expected to be negligible. However, the absence of pharmacokinetic studies on ricinoleic acid in lactating women leaves gaps in understanding its excretion into breast milk. Additionally, no data are available on the metabolism of ricin or ricine in lactating women, though these toxic components are largely removed during oil production.

Relevant studies on the effects of castor oil use during breastfeeding are sparse, and no published data detail its excretion into breast milk or its impact on breastfed infants. Traditional practices in rural India have involved administering castor oil to newborns during the first days of life to clear meconium. These practices have led to severe complications such as paralytic ileus and aspiration pneumonia [128]. In one case, a 1.5-month-old infant developed severe hypoalbuminemia, diarrhea, and malnutrition after being given castor oil daily from the fifth day of life, which illustrates the potential dangers of castor oil use in infants [129]. While these outcomes resulted from castor oil direct administration to infants, the risks associated with the maternal use of this substance during breastfeeding remain speculative due to insufficient evidence.

No scientific evidence supports the use of castor oil as a galactagogue. Although traditional claims suggest that castor oil or castor leaf poultices enhance milk production, no clinical trials have validated these assertions. Additionally, the safety of castor oil or its components when used topically or orally by lactating mothers is unknown. Given the potential for toxicity and the lack of data on efficacy, alternative approaches to improving milk supply should be prioritized. Counseling on proper breastfeeding techniques and addressing modifiable factors affecting milk production remain the gold standard [109,110].

Castor oil has a long history of traditional use in various cultures as both a cathartic and a purported galactagogue. However, no scientific evidence supports its effectiveness in increasing milk supply, and its safety profile for breastfeeding mothers and infants is poorly defined. Ricinoleic acid, the active component of castor oil, has minimal systemic absorption, suggesting limited transfer into breast milk. Nevertheless, the absence of robust pharmacokinetic and clinical data precludes its recommendation for lactating women. Given the potential risks associated with castor oil use, alternative, evidence-based approaches to managing lactation issues are strongly advised. Further research is needed to clarify the safety and efficacy of castor oil and its derivatives in breastfeeding populations.

### 6.3. Caffeine

Caffeine is a stimulant commonly found in coffee, tea, cola, energy drinks, and certain medications. It appears rapidly in breast milk after maternal ingestion, with insufficient high-quality data available to establish definitive guidelines for safe maternal caffeine consumption during lactation (1). Infants of mothers with very high caffeine intakes (equivalent to 10 or more cups of coffee daily) exhibited symptoms such as fussiness, jitteriness, and disrupted sleep patterns. However, studies indicate that moderate maternal caffeine consumption, such as five cups of coffee daily, does not appear to stimulate breastfed infants aged 3 weeks or older. A daily maternal intake limit of 300 to 500 mg of caffeine may be considered safe for most mothers, while European authorities recommend a lower limit of 200 mg [130].

Preterm and younger newborn infants metabolize caffeine very slowly due to immature liver enzymes, resulting in serum caffeine levels similar to those in their mothers. This slower metabolism suggests that the mothers of preterm or younger infants should limit caffeine intake further [131,132]. Other caffeine sources, including yerba mate, guarana, and certain medications, may have similar dose-related effects on breastfed infants. Additionally, a maternal coffee intake exceeding 450 mL daily may lower breast milk iron concentrations and result in mild iron-deficiency anemia in some breastfed infants [133].

Caffeine is primarily metabolized in the liver by cytochrome P450 1A2 (CYP1A2) into three active metabolites: paraxanthine, theobromine, and theophylline. These metabolites are further processed through demethylation and oxidation pathways. During pregnancy, the half-life of caffeine significantly increases due to reduced CYP1A2 activity, but this enzyme’s activity returns to normal postpartum [134].

In smokers, CYP1A2 is induced, leading to faster clearance of caffeine. In contrast, preterm and newborn infants have immature liver enzyme systems and extremely low caffeine clearance rates, which normalize by 3 to 5 months of age [131]. Caffeine’s elimination half-life in breastfeeding mothers is similar to that in nonpregnant adults, typically ranging from 3 to 7 h [134]. Caffeine transfers into breast milk, with peak concentrations occurring about 1 h post-ingestion [135,136,137]. Active metabolites are also present in breast milk, but their concentrations peak later, with paraxanthine, theobromine, and theophylline reaching their highest levels between 5 and 15 h after maternal ingestion [132].

Several studies have examined maternal caffeine intake and its impact on breast milk concentrations and infant exposure. A study of five women 4 months to 1 year postpartum found that a single dose of 150 mg of caffeine resulted in peak breast milk levels of 1.6 mg/L at 30 min, declining to 0.9 mg/L by 120 min [138]. Another study of two mothers at 7 and 13 weeks postpartum who consumed 128 mg of caffeine recorded peak milk levels of 1.3 to 1.6 mg/L within 2 h [136].

Mothers who consumed higher caffeine doses showed proportional increases in milk caffeine concentrations. For example, the pooled milk samples from nine women consuming 750 mg of caffeine daily over 5 days averaged 4.3 mg/L, with some milk samples reaching up to 28.6 mg/L. By the fourth day of caffeine abstinence, no detectable caffeine remained in breast milk [139]. Additionally, a randomized, double-blind study in 11 women who consumed 500 mg of caffeine daily for 5 days found average milk caffeine concentrations of 3.1 mg/L, resulting in infant intakes of approximately 0.5 mg/kg daily [130].

The variability in caffeine levels in milk corresponds to maternal caffeine intake, timing of ingestion, and individual metabolic differences. In one case, a mother’s single cup of espresso resulted in a peak milk caffeine concentration of 2.05 ng/L after 2 h, with undetectable levels after 24 h. Her infant consumed an estimated 0.1 mg/kg of caffeine daily, representing 8.9% of her weight-adjusted dosage [140].

Infants exposed to caffeine via breast milk demonstrate dose-related effects. One study noted jitteriness and increased muscle tone in a 6-week-old infant whose mother consumed four to five cups of coffee and several bottles of cola daily. The symptoms resolved two weeks after cessation of maternal caffeine intake [141]. Another case involved a breastfed 5-month-old whose mother consumed 20 cups of coffee daily. The infant exhibited restlessness and irritability until maternal caffeine intake was reduced [142].

However, studies in mothers with moderate caffeine consumption, such as up to 500 mg daily, found no significant effects on infant heart rate or sleep duration. For example, a cohort study involving low-income mothers in Costa Rica reported lower hemoglobin levels in infants of heavy coffee drinkers [133]. Additionally, a randomized trial examining caffeine exposure through decaffeinated coffee with added caffeine showed no adverse effects on infant sleep time or behavior [130].

Limited data are available on the direct effects of caffeine on lactation physiology. Excessive caffeine intake may reduce breast milk iron concentrations, potentially contributing to mild anemia in breastfed infants [133]. However, no studies have identified significant impacts of caffeine on milk production or composition.

Caffeine rapidly transfers into breast milk, with peak levels occurring within 1 to 2 h post-ingestion. While a moderate maternal caffeine intake appears safe for most breastfed infants, high consumption may cause symptoms such as irritability and poor sleep, particularly in preterm or younger infants with slower caffeine metabolism. A daily maternal caffeine intake of 300 to 500 mg is generally considered safe, though a lower limit of 200 mg is advisable for mothers of preterm infants. Further research is needed to fully understand the long-term effects of caffeine exposure in breastfeeding populations.

### 6.4. Methamphetamine

Methamphetamine is metabolized to amphetamine, impacting breast milk concentrations. When methamphetamine is ingested, it is absorbed into the bloodstream and distributed throughout the body. The liver metabolizes it primarily through the enzyme CYP2D6, converting it into amphetamine, which is also active, prolonging the drug’s effects. Both methamphetamine and its metabolites are excreted in the urine [143]. The half-life of methamphetamine is approximately 10 h, but this can vary based on factors like the pH level of urine, which can affect the rate of excretion [144]. Methamphetamine can pass into breast milk and may have harmful effects on a nursing infant. Infants exposed to methamphetamine through breast milk can experience irritability, poor feeding, and hypertension. Due to these risks, healthcare providers generally advise against breastfeeding while using methamphetamine. Mothers using this drug are encouraged to discuss safer alternatives and treatment options with their healthcare provider to ensure the well-being of their child.

In studies of mothers who abused methamphetamine, the peak milk concentrations varied significantly. One mother had levels of 160 mcg/L, while another reached 610 mcg/L. The average concentrations were 111 mcg/L and 281 mcg/L, respectively, with half-lives of 13.6 and 7.4 h. The amphetamine levels were relatively constant, averaging 4 and 15 mcg/L. The estimated infant doses were 16.7 to 42.2 mcg/kg of methamphetamine and 0.8 to 2.5 mcg/kg of amphetamine daily [145]. This data, however, are not generalizable to all methamphetamine abusers. In Thailand, 33 mothers with positive drug screens revealed varying milk concentrations. While 22 had undetectable levels, two mothers had significant levels, with methamphetamine half-lives in milk of 11.3 and 40.3 h. Methamphetamine in milk became undetectable around 100 h post-use [146]. Another analysis found varying concentrations in suspected abusers’ milk, with methamphetamine levels ranging from 3.8 to 327 mcg/L [147].

Cases have been reported where methamphetamine exposure through breastfeeding may have contributed to infant deaths, although causality is debated due to low serum levels and minimal breastfeeding [148,149]. Concerns arise from significant methamphetamine findings in autopsy cases, suggesting potential risks [149].

Methamphetamine and amphetamines can affect the prolactin levels. A single oral dose of d-methamphetamine did not alter the serum prolactin levels in non-lactating individuals [150]. However, dextroamphetamine reduced the prolactin levels postpartum, potentially affecting milk production, though no direct assessment of milk output was made [151,152]. Methamphetamine-dependent mothers were less likely to breastfeed at discharge, and their infants showed altered feeding behaviors compared to non-exposed infants [153].

Methamphetamine in breast milk poses significant risks due to its variable concentrations and potential infant exposure. While some infants show no immediate effects, the drug’s impact on infant health and development is concerning, especially at high exposures. Methamphetamine use is associated with reduced breastfeeding rates and potential alterations in infant behavior, highlighting the need for caution and support for affected mothers. Alternative treatments and interventions should be considered to ensure infant safety.

### 6.5. Cocaine

Cocaine is a potent stimulant with a high potential for harm, especially when transferred to breastfed infants through breast milk. The drug is lipophilic, allowing significant concentrations to pass into breast milk. Adverse effects on infants can range from irritability and feeding difficulties to severe neurological and cardiovascular symptoms. Given the variability in reported cocaine concentrations in breast milk and its highly toxic nature, breastfeeding mothers are strongly advised against using cocaine. Additionally, smoking “crack” cocaine near infants is particularly dangerous due to the risk of passive inhalation. When cocaine is used occasionally, a breastfeeding abstinence period of at least 24 h is recommended to minimize infant exposure. Legal and ethical concerns may also arise if cocaine or its metabolites are found in an infant’s urine, further emphasizing the importance of avoiding cocaine use during breastfeeding [154,155,156].

Cocaine undergoes extensive hepatic metabolism primarily via butyrylcholinesterase and carboxylesterase 1, forming major metabolites such as benzoylecgonine, ecgonine methyl ester, and norcocaine. These metabolites are detectable in breast milk alongside unmetabolized cocaine. When combined with alcohol, cocaethylene is formed, which has a longer half-life and enhanced toxicity. The elimination of cocaine and its metabolites from the mother’s body is rapid, with most substances becoming undetectable in breast milk within 24 to 36 h post-ingestion. However, variability exists depending on factors like dose, route of administration, and maternal metabolism [155,157,158].

One case involved a mother who reported the intranasal use of 500 mg of cocaine over a 4 h period. She breastfed her 2-week-old infant five times during this time. The infant began exhibiting symptoms three hours after the first breastfeeding, including severe irritability, vomiting, dilated pupils, and diarrhea. Upon examination in the emergency room, the infant was noted to have tremors, high-pitched crying, mood instability, and hypertension. Urine analysis revealed cocaine concentrations of 100 mcg/L at 4 and 12 h post-ingestion, and detectable levels persisted up to 60 h after the mother’s last use. The infant’s symptoms resolved gradually over 72 h [155].

Another severe case involved an 11-day-old infant whose mother applied cocaine powder directly to her nipples to relieve pain before breastfeeding. Three hours after feeding, the infant was found cyanotic and gasping for air. In the emergency room, the infant exhibited hypertension, tachycardia, shallow breathing, and seizures. Toxicological screening confirmed cocaine exposure. The infant recovered after several hours of treatment and was discharged without apparent long-term effects. This case illustrates the extreme risks of direct exposure to cocaine [159].

A third case documented a 1-month-old infant brought to the emergency department with hypoactivity, seizures, and nose bleeding. The mother admitted to regular cocaine and marijuana use. Toxicology tests revealed cocaine in the infant’s plasma. The infant was hospitalized for four days, during which two seizures were observed. The symptoms were attributed to cocaine withdrawal, highlighting the potential for serious health risks even in the absence of direct breastfeeding exposure.

In a study conducted in a Brazilian hospital, five mothers suspected of cocaine use provided breast milk samples. The cocaine concentrations ranged from 24.4 to 38.5 mcg/L, while the benzoylecgonine levels varied between 17.6 and 91.2 mcg/L. The variability in the metabolite levels suggests differences in the timing and dosage of cocaine use relative to milk collection. These findings underscore the unpredictability of cocaine transfer into breast milk and its potential impact on exposed infants [160].

Cocaine is highly toxic to infants due to their immature metabolic systems, which limit their ability to detoxify the drug. Infants exposed to cocaine via breast milk or passive inhalation may experience severe symptoms, including irritability, seizures, and cardiovascular instability. The significant variability in cocaine and metabolite concentrations in breast milk makes it difficult to predict infant exposure accurately. Recommendations include avoiding cocaine use during breastfeeding entirely and observing a minimum 24 h abstinence period after occasional use. Awareness of the potential legal and medical consequences further underscores the importance of avoiding cocaine use while breastfeeding [155,156,159,160].

### 6.6. Alcohol

Alcohol use during breastfeeding presents a complex interplay of risks to both maternal lactation and infant development. Alcohol transfers rapidly into breast milk, with concentrations closely paralleling the maternal blood alcohol levels [161,162]. The timing and quantity of alcohol consumption are critical, as even a moderate intake can reduce milk production, disrupt the milk ejection reflex, and alter infant feeding behaviors [163,164,165,166]. In infants, alcohol exposure through breast milk has been associated with reduced milk intake, disrupted sleep cycles, and, in cases of chronic maternal use, potential developmental delays or growth impairments [40,161,167]. Although casual, well-timed alcohol consumption may pose minimal risks when breastfeeding is delayed appropriately post-consumption, its heavy or frequent use has been linked to long-term consequences, including reduced motor skills and slower growth rates [168,169,170].

Alcohol (ethanol) is rapidly absorbed into the bloodstream through the gastrointestinal tract, with peak blood concentrations typically occurring 30 to 60 min after ingestion, though this can be delayed by food intake [161,162]. Once in the bloodstream, alcohol is metabolized primarily in the liver through enzymatic pathways involving alcohol dehydrogenase (ADH) and aldehyde dehydrogenase (ALDH). These enzymes convert alcohol to acetaldehyde, a toxic intermediate, and subsequently to acetate, which is further metabolized to carbon dioxide and water [171]. The rate of alcohol metabolism can vary based on genetic polymorphisms, enzyme activity, and other individual factors such as sex and age [161,165]. The elimination half-life of alcohol is approximately 2 to 3 h in most individuals, but this can vary depending on factors like body weight, liver function, and concurrent food intake [165,172]. During this period, alcohol can transfer into breast milk, with concentrations closely paralleling the maternal blood alcohol levels [161,162]. Importantly, alcohol is not stored in milk, but its levels in milk equilibrate with the mother’s blood alcohol content, meaning that as the maternal blood alcohol levels decline, so do the milk alcohol levels [172]. For breastfeeding mothers, the timing of alcohol consumption is critical. Studies suggest that waiting 2 to 2.5 h per standard drink (containing approximately 12 g of alcohol) allows sufficient time for alcohol clearance from the bloodstream and breast milk [172]. However, heavy or frequent alcohol consumption poses greater risks, including reduced milk production, altered milk ejection reflex, and potential developmental impacts on the nursing infant [163,164,165]. Infants exposed to alcohol through breast milk may experience irritability, sleep disturbances, and altered feeding patterns [40,167]. Given these metabolic and transfer characteristics, mothers are advised to minimize alcohol use while breastfeeding and follow evidence-based guidelines to ensure infant safety. For heavy alcohol consumption, expressed milk should be discarded until the alcohol levels have normalized [173].

Studies examining the impact of maternal alcohol consumption on breastfeeding and infant health have provided critical insights. Alcohol transfers into breast milk rapidly, with milk concentrations closely paralleling the maternal blood alcohol levels [161,162]. A study involving lactating women found that consuming 0.3 g/kg of alcohol resulted in peak milk alcohol concentrations ranging from 0.32 to 1.05 g/L, depending on individual factors like food intake and body weight [40,174]. In another study, the milk alcohol levels became negligible around three hours post-ingestion, highlighting the temporal nature of alcohol’s presence in breast milk [172]. Breastfed infants exposed to alcohol-containing milk exhibit significant behavioral changes, including reduced milk intake by 20–23%, disrupted sleep patterns, and increased crying episodes [40,175]. These effects are thought to be linked to altered milk flavor and reduced caloric intake. Long-term exposure to alcohol may contribute to developmental delays, including reduced motor function, though the cognitive impacts are less consistent [168,169]. A study comparing infants exposed to alcohol during breastfeeding with infants who were not found no differences in mental development at 18 months, though some motor delays were noted at 1 year.

In rare cases, a heavy maternal alcohol use has been associated with severe infant outcomes. For example, the infants of mothers who consumed large quantities of alcohol postpartum developed pseudo-Cushing syndrome, characterized by excessive weight gain and bloated appearance, which resolved upon cessation of maternal alcohol intake [176]. Another report described a neonate experiencing deep, unarousable sleep after the mother consumed 750 mL of Port wine in 24 h, indicating alcohol’s sedative effects [177].

Alcohol consumption can also influence lactation physiology. Studies indicate that alcohol reduces oxytocin release, delaying the milk ejection reflex. Higher doses exacerbate this effect, with doses of 1.5 g/kg delaying milk letdown to as long as 331 s [163,164,165]. Additionally, alcohol ingestion has been linked to lower milk production, with a 0.3 g/kg dose reducing the milk output by 9.3% in lactating women [166].

Alcohol-based cultural practices during lactation also vary globally. For instance, postpartum consumption of Chicken wine has been associated in Chinese culture with ileus and abdominal distension in breastfed infants, highlighting potential culture-specific risks [178]. Similarly, Mexican mothers consuming pulque, an alcohol-containing beverage, displayed mixed outcomes in infant growth and health, with some studies noting poorer growth trajectories compared to non-exposed infants [179,180].

Alcohol consumption during breastfeeding carries potential risks for both maternal lactation and infant development. The rapid transfer of alcohol into breast milk, with its levels paralleling the maternal blood alcohol levels, underscores the importance of timing and moderation for its consumption. Heavy alcohol use can disrupt milk ejection, reduce its production, and cause adverse infant outcomes, including altered feeding patterns, sleep disturbances, and developmental delays. Cultural practices involving alcohol highlight variable impacts across populations, emphasizing the need for context-specific guidance. Mothers are strongly encouraged to minimize their alcohol intake, adhere to safe breastfeeding practices, and seek professional support when necessary to ensure the health and safety of their infants.

## 7. Cannabis in Breastfeeding

### 7.1. Tetrahydrocannabinol

Tetrahydrocannabinol (THC), the primary psychoactive component of cannabis, is excreted into breastmilk in small amounts and can persist in milk for extended periods. THC has been quantified in milk for 6 days to over 6 weeks after maternal use, depending on frequency and intensity of its consumption [181,182]. Its estimated half-life in milk is between 12 and 39 h, allowing clearance within 2.5 to 8 days post-exposure. Concerns about cannabis use during lactation focus on its potential effects on infant nervous system development, neurotransmitter function, and the endocannabinoid system [183,184]. Studies suggest that daily or near-daily maternal use may slightly retard infant motor development at one year of age, though no significant effects on intellectual development or growth were reported [185]. Occasional maternal cannabis use during lactation has not shown discernable effects, but these findings are based on earlier studies involving cannabis with lower potency [186].

Cannabis use may reduce the serum prolactin levels and subsequently decrease milk supply, potentially shortening the duration of breastfeeding [187,188]. Additionally, cannabis users have demonstrated altered milk composition, with increases in protein and carbohydrate concentrations, while the fat and energy levels remain unaffected [189]. Current professional guidelines advise mothers to avoid cannabis use during lactation to mitigate the potential adverse effects on their infants [187,190].

THC undergoes metabolism primarily in the liver via cytochrome P450 enzymes, notably CYP2C9 and CYP3A4. These enzymes convert THC to its active metabolite, 11-hydroxy-THC, which is further oxidized to inactive 11-carboxy-THC [191]. THC’s high lipophilicity leads to its sequestration in adipose tissues, allowing for its slow redistribution into the circulation and breastmilk over days or weeks [181]. Breastmilk THC concentrations are influenced by maternal factors, including frequency of use and route of administration. Milk-to-plasma ratios have been reported at 0.4 to 8.7%, indicating substantial variability among users [192].

Two lactating women who smoked cannabis daily provided randomly collected breastmilk samples for THC analysis. The first mother, who smoked once daily, exhibited a breastmilk THC concentration of 105 mcg/L, while the second mother, who smoked 7–8 times daily, had a concentration of 340 mcg/L. In addition, a milk sample collected one-hour post-smoking showed THC levels of 60.3 mcg/L, along with 1.1 mcg/L of 11-hydroxy-THC and 1.6 mcg/L of 9-carboxy-THC [181]. This indicates significant variability in THC levels, likely influenced by the frequency and timing of cannabis use. Based on these data, infant exposure was estimated at 0.8% of the maternal weight-adjusted dosage [181,182].

Eight exclusively breastfeeding women consumed 100 mg of cannabis containing 23.18% THC after a 24 h abstention period. Breastmilk was collected at multiple intervals, including at baseline, 20 min, 1, 2, and 4 h post-inhalation. Six participants had undetectable baseline THC (<2 mcg/L), while two had detectable levels of 5.8 mcg/L and 15.8 mcg/L. Post-inhalation, the median breastmilk THC concentration was 27.6 mcg/L, peaking at 94 mcg/L one hour after smoking. The individual peak levels varied widely, ranging from 12.2 to 420.3 mcg/L. Active metabolites, including 11-hydroxy-THC and 11-nor-9-carboxy-THC, were below the detection thresholds (<0.097 mcg/L) in all samples (18). The estimated daily THC intake for infants was approximately 8 mcg/kg, corresponding to 2.5% (range: 0.4–8.7%) of the maternal weight-adjusted dosage [192].

Fifty lactating women who reported cannabis use within the prior 14 days provided 54 breastmilk samples. THC was detectable in 63% of the samples, with a median concentration of 9.47 mcg/L (range: 1–323 mcg/L). Among these, only five samples contained measurable levels of 11-hydroxy-THC, ranging from 1.3 to 12.8 mcg/L. Breastmilk collected 140 h or more after cannabis use showed undetectable THC levels (<1 mcg/L). The estimated half-life of THC in breastmilk was approximately 27 h, and infant exposure was calculated at an average of 1.4 mcg/kg per day [193].

In a study of 20 lactating women, cannabis was consumed daily by smoking or ingestion. The median breastmilk THC concentrations were 27.5 mcg/L at two weeks postpartum and 54.5 mcg/L at two months postpartum, indicating an increase with continued use. The estimated daily THC intake for infants ranged from 0.52 to 123 mcg/kg, with a median of 4.12 mcg/kg [194]. The THC levels were consistent across consumption methods (smoking versus edibles). Milk samples from chronic users revealed a median elimination half-life of THC in breastmilk of 11.45 h. The presence of metabolites, including 11-hydroxy-THC and carboxy-THC, indicated active metabolic processing [195].

A 6-month-old breastfed infant whose mother was a chronic cannabis user presented with somnolence and seizure-like symptoms. Blood and urine analyses revealed carboxy-THC concentrations of 189 mcg/L and 423 mcg/L, respectively. The infant recovered fully within 72 h after cessation of breastfeeding [196].

In another case, a 9-month-old girl experienced tonic–clonic seizures and had delta-9-THC (2.2 mcg/L), delta-9-THC-COOH (1.1 mcg/L), and 11-OH-THC (0.4 mcg/L) determined in her plasma. Her mother reported smoking multiple joints daily while breastfeeding. The symptoms resolved after breastfeeding was discontinued [197].

A physiologically based pharmacokinetic (PBPK) model estimated infant plasma THC levels based on maternal use. For mothers smoking cannabis six times daily, the predicted infant plasma THC concentrations ranged from 0.084 to 0.167 mcg/L, significantly lower than the maternal plasma levels. Neonates had the highest predicted THC exposure due to immature enzyme activity and slower metabolism [198].

Cannabis use during lactation presents risks due to THC’s prolonged presence in breastmilk and its potential effects on infant development and maternal lactation. While occasional maternal use appears to pose minimal short-term risks, daily or heavy consumption has been linked to motor development delays, somnolence, and seizure-like activity in infants [185]. THC’s lipophilic nature, slow clearance, and active metabolites emphasize the importance of avoiding cannabis during breastfeeding. Healthcare providers should educate mothers on these risks and provide resources for cessation support, prioritizing infant safety and developmental outcomes [187,190].

### 7.2. Cannabidiol

Cannabidiol (CBD), a non-psychoactive component of cannabis, has been determined in breastmilk of mothers who use cannabis products. Due to its high lipophilicity, CBD readily partitions into fatty tissues, including breastmilk. While it has therapeutic potential, including as an antiepileptic, the absence of clinical safety data on its use during breastfeeding raises concerns. The predicted infant exposure from breastmilk appears minimal, typically less than 1% of the therapeutic pediatric dose. Nevertheless, the potential for cumulative exposure and unknown long-term effects suggest that alternative treatments should be preferred for nursing mothers, especially those with newborns or preterm infants [193,199,200].

CBD has been quantified in breastmilk at concentrations influenced by the method of ingestion. In one study, the median CBD concentration in milk samples was 5 mcg/L (range: 1.3–8.6 mcg/L) [193]. A physiologically based pharmacokinetic (PBPK) model analyzed 200 samples from 181 mothers and showed higher concentrations in milk when CBD was ingested via oil or a pipe compared to edibles or joints. The median concentration for oil ingestion was 6.4 mcg/L (range: 2.1–9.5 mcg/L), while edibles yielded a median concentration of 4.7 mcg/L (range: 1.3–7.8 mcg/L) [200].

CBD is metabolized in the liver primarily by CYP3A4 and CYP2C19, producing active metabolites like 7-hydroxy-CBD (7-OH-CBD) and inactive metabolites such as 7-carboxy-CBD (7-COOH-CBD). Its lipophilicity causes it to accumulate in fatty tissues, contributing to its persistence in the body. The elimination half-life of CBD in breastmilk is approximately 18.7 h (range: 12–27 h), and peak concentrations in milk generally occur 2–4 h post-ingestion for oil and pipe use [200].

In a PBPK model study, infant exposure to CBD through breastmilk was projected to be less than 1% of the therapeutic dose for pediatric epilepsy. For mothers consuming CBD oil, the estimated infant dose was 0.5–0.8 mcg/kg/day, with the corresponding plasma levels in the infant remaining below 0.05 mcg/L. In comparison, edibles resulted in slightly lower concentrations in milk but still minimal predicted exposure for the infant [200].

While limited data exist, no adverse effects in breastfed infants have been reported in connection with maternal CBD use. The absence of detectable cannabidiol in the plasma of breastfed infants in most studies further supports the conclusion that infant exposure is likely negligible. However, the cumulative effects of low-level, chronic exposure remain unknown [200].

Cannabidiol’s lipophilic nature and hepatic metabolism result in detectable but low concentrations in breastmilk, particularly following oil or pipe ingestion. Although predicted infant exposure is minimal, the absence of comprehensive safety studies on CBD during breastfeeding warrants caution. Until further evidence becomes available, healthcare providers should consider recommending alternative medications for nursing mothers, especially for those caring for newborns or preterm infants.

## 8. Pharmacokinetics: Relative Infant Dose (RID) and Milk-To-Plasma (MP) Ratio

The MP ratio is a measure used to determine the extent to which a drug transfers from maternal plasma into breast milk [201]. It is calculated by dividing the concentration of the drug in breast milk by its concentration in maternal plasma. Its advantages are simplicity of calculation, provision of a straightforward estimate of drug transfer into milk, and initial-screening usefulness for quickly assessing whether a drug is likely to be present in significant amounts in breast milk. As for its drawbacks, its variability is low, as it can vary depending on the timing of sample collection relative to drug dosing, and it does not account for total exposure of the infant or the pharmacokinetics of the drug within the infant.

The calculator formula is shown below:

[MP Ratio] = [Concentration of drug in milk]/[Concentration of drug in plasma]

The relative infant dose (RID) is a key pharmacological metric used to assess the safety of medication exposure for breastfeeding infants. It is defined as the percentage of the maternal dose that is transferred to the infant through breast milk. The formula for calculating the RID is as follows:

[RID] = [Infant dose via milk] (mL/kg/day)/[Maternal dose] (mL/kg/day)

To analyze the RID, first determine the infant’s dose by measuring the concentration of the drug in breast milk and estimating the volume of milk consumed by the infant per day [202]. Then, calculate the maternal dose based on the mother’s weight and the medication dosage she receives. An RID of less than 10% is generally considered safe, suggesting minimal risk to the infant. However, specific safety thresholds can vary depending on the drug’s properties and the infant’s health. Sometimes, a lower value of RID such as 5% can also be considered as a reference point. A comprehensive evaluation should include consideration of the drug’s pharmacokinetics, the infant drug clearance, and potential effects on the infant.

Table 1 summarizes maternal drug metabolites and weight-adjusted dosages in breast milk, with sedatives being the most extensively studied category. Among sedatives, diazepam (3%), alprazolam (3%), and lorazepam (8.5%) exhibit low-to-moderate maternal weight-adjusted dosages, while phenobarbital stands out with an exceptionally high dosage of 72.5%. Haloperidol lacks reported dosage data, despite its glucuronidation metabolites being detectable. Other sedatives, such as dichloralphenazone (0.59%) and propofol derivatives (0.2–0.2%), show minimal transfer. Inhalants like methamphetamine and substances such as cannabis cannabidiol, cocaine, and castor oil lack dosage data entirely, despite producing detectable metabolites like amphetamine or ricinoleic acid. Stimulants and psychoactive agents, including sulpiride (2.0–18.0%) and caffeine (10.0–18.0%), display moderate-to-high variability, whereas nicotine (1.9%) and alcohol (0.5–3.3%) show lower ranges of weight-adjusted dosages. Notably, cannabis exhibits a broad dosage range (0.4–8.7%) for its primary metabolite 11-OH-THC, which contrasts with the absence of reported dosage for cannabidiol. Overall, the available data for sedatives are dominant, while significant gaps persist for certain substances, particularly non-sedative classes.

**Figure 2:**
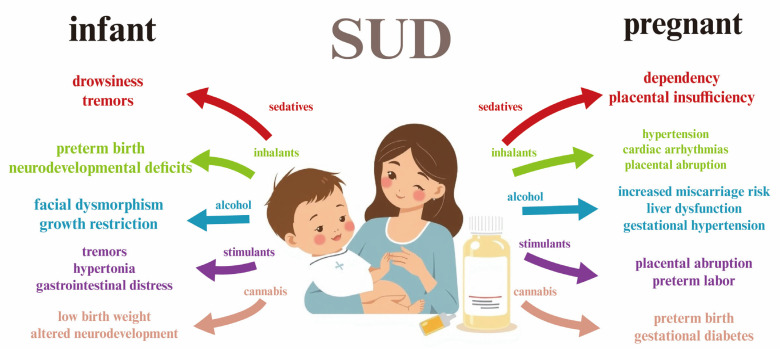
SUD effects on pregnant and infant populations.

Substance use disorder (SUD) during pregnancy and lactation poses significant risks to both maternal and infant health, as highlighted in Figure 2. Prenatal exposure to substances like sedatives, alcohol, stimulants, inhalants, tobacco, and cannabis is linked to complications such as placental insufficiency, miscarriage, preterm birth, and gestational hypertension in mothers. For infants, these exposures can lead to neurodevelopmental deficits, growth restriction, tremors, and low birth weight. Postnatally, many substances can be transmitted through breast milk, potentially causing sedation, irritability, poor feeding, or delayed development in nursing infants. Due to infants’ immature metabolism, drug exposure through breastfeeding can lead to prolonged effects. Therefore, providing counseling and treatment for individuals with SUD is crucial to minimizing harm to both mother and child.

## 9. Conclusions

This paper explored the multifaceted role of breast milk in infant development and the complexities of drug transfer during lactation. Breast milk offers crucial benefits, including reduced infection rates and enhanced cognitive development in infants, attributed to components like secretory IgA and lactoferrin. These benefits are especially pronounced in preterm infants, where breastfeeding significantly lowers the risk of necrotizing enterocolitis.

This study delved into substance use disorder (SUD) during pregnancy, a condition that poses significant risks to both mother and fetus. SUD can lead to adverse outcomes such as low birth weight and developmental disorders, with different substances impacting fetal health in varying ways. The paper discussed the importance of understanding these impacts to mitigate risks to infants.

Finally, the paper addressed the cautious use of sedatives in breastfeeding mothers, emphasizing the need for limited duration use and close monitoring of infants to avoid adverse effects. This comprehensive analysis aims to guide healthcare providers in balancing the benefits of breastfeeding with the potential risks associated with maternal drug use.

Despite compiling a broad range of evidence on the transfer of substances into breast milk and their potential effects on infants, several limitations should be acknowledged.

First, although this review covers multiple commonly used substances such as sedatives, stimulants, inhalants, tobacco, alcohol, and cannabis, the current body of evidence is largely limited to case reports or small observational studies. Most existing studies lack a systematic experimental design. Randomized controlled trials, prospective cohort studies with predefined endpoints, and standardized sampling schedules are largely absent. Instead, the available evidence is often derived from retrospective reviews or uncontrolled observations, which are insufficient to establish causality. Moreover, the small sample sizes and lack of standardization in milk sampling protocols hinder the generalizability of these findings. Additionally, important pharmacokinetic parameters such as milk-to-plasma (M/P) ratio and relative infant dose (RID) are not consistently reported or calculated, limiting the ability to quantitatively make comparisons across studies.

In addition, critical confounding factors—such as maternal disease severity, concurrent medication use, and variability in dosage or timing—are frequently unaccounted for. The current documentation of adverse drug reactions (ADRs) in breastfed infants is primarily based on isolated case reports, which often lack detailed pharmacokinetic data. Without rigorous design and stratification, it is difficult to isolate the specific impact of drug exposure through breast milk on infant outcomes.

To address these gaps, future studies should employ structured pharmacokinetic designs—including physiologically based pharmacokinetic (PBPK) models tailored to lactating mothers and infants—and should include prospective infant monitoring. These approaches can provide more mechanistic and quantitative insight into maternal–infant drug transfer and support more evidence-based breastfeeding recommendations.

## Figures and Tables

**Table 1:** Maternal drug metabolites and weight-adjusted dosage in breast milk.

Type	Name	Metabolites	Maternal Weight-Adjusted Dosage (%)
Sedative	Diazepam	nordiazepam, temazepam, and oxazepam	3
	Phenobarbital	*p*-hydroxyphenobarbital	72.5
	Haloperidol	glucuronidation	None
	Dichloralphenazone	chloral hydrate, trichloroethanol and trichloroacetic acid	0.59
	Fospropofol	propofol	0.2
	Alprazolam	alpha-hydroxyalprazolam	3
	Propofol	propofol-glucuronide and sulfo- and glucuro-conjugation	0.2
	Carisoprodol	meprobamate	6–6.9
	Lorazepam	glucuronide conjugate	8.5
Inhalant	Methamphetamine	amphetamine	None
Tobacco	Nicotine	cotinine	1.9
Alcohol	Alcohol	acetaldehyde and acetate	0.5–3.3
Stimulant	Sulpiride	desmethylsulpiride	2.0–18.0
	Castor oil	ricinoleic acid and glycerol	None
	Caffeine	paraxanthine and theobromine	10.0–18.0
Cannabis	Cannabis	11-OH-THC and glucuronide conjugates	0.4–8.7
	Cannabidiol	7-OH-CBD and glucuronide conjugates of CBD metabolites	None
	Cocaine	benzoylecgonine, ecgonine methyl ester, and cocaethylene	None

## Data Availability

The data that support the findings of this study are available from the corresponding author upon reasonable request.
